# Association of ultra-processed food consumption with colorectal cancer risk among men and women: results from three prospective US cohort studies

**DOI:** 10.1136/bmj-2021-068921

**Published:** 2022-08-31

**Authors:** Lu Wang, Mengxi Du, Kai Wang, Neha Khandpur, Sinara Laurini Rossato, Jean-Philippe Drouin-Chartier, Euridice Martínez Steele, Edward Giovannucci, Mingyang Song, Fang Fang Zhang

**Affiliations:** 1 Friedman School of Nutrition Science and Policy, Tufts University, Boston, MA, USA; 2 Department of Epidemiology, Harvard T.H. Chan School of Public Health, Boston, MA, USA; 3 Department of Nutrition, School of Public Health, University of São Paulo, São Paulo, Brazil; 4 Center for Epidemiological Studies in Health and Nutrition (NUPENS), Faculty of Public Health, University of São Paulo, Brazil; 5 Department of Nutrition, Harvard T.H. Chan School of Public Health, Boston, MA, USA; 6 Institute of Geography, Universidade Federal de Uberlândia, Minas Gerais, Brazil; 7 Centre Nutrition, Santé et Société (NUTRISS), Institut sur la Nutrition et les Aliments Fonctionnels (INAF), Faculté de Pharmacie, Université Laval, Québec, QC, Canada; 8 Channing Division of Network Medicine, Department of Medicine, Brigham and Women’s Hospital, Boston, MA, USA; 9 Clinical and Translational Epidemiology Unit, Massachusetts General Hospital and Harvard Medical School, Boston, MA, USA; 10 Division of Gastroenterology, Massachusetts General Hospital and Harvard Medical School, Boston, MA, USA

## Abstract

**Objective:**

To examine the association between consumption of ultra-processed foods and risk of colorectal cancer among men and women from three large prospective cohorts.

**Design:**

Prospective cohort study with dietary intake assessed every four years using food frequency questionnaires.

**Setting:**

Three large US cohorts.

**Participants:**

Men (n= 46 341) from the Health Professionals Follow-up Study (1986-2014) and women (n=159 907) from the Nurses’ Health Study (1986-2014; n=67 425) and the Nurses’ Health Study II (1991-2015; n=92 482) with valid dietary intake measurement and no cancer diagnosis at baseline.

**Main outcome measure:**

Association between ultra-processed food consumption and risk of colorectal cancer, estimated using time varying Cox proportional hazards regression models adjusted for potential confounding factors.

**Results:**

3216 cases of colorectal cancer (men, n=1294; women, n=1922) were documented during the 24-28 years of follow-up. Compared with those in the lowest fifth of ultra-processed food consumption, men in the highest fifth of consumption had a 29% higher risk of developing colorectal cancer (hazard ratio for highest versus lowest fifth 1.29, 95% confidence interval 1.08 to 1.53; P for trend=0.01), and the positive association was limited to distal colon cancer (72% increased risk; hazard ratio 1.72, 1.24 to 2.37; P for trend<0.001). These associations remained significant after further adjustment for body mass index or indicators of nutritional quality of the diet (that is, western dietary pattern or dietary quality score). No association was observed between overall ultra-processed food consumption and risk of colorectal cancer among women. Among subgroups of ultra-processed foods, higher consumption of meat/poultry/seafood based ready-to-eat products (hazard ratio for highest versus lowest fifth 1.44, 1.20 to 1.73; P for trend<0.001) and sugar sweetened beverages (1.21, 1.01 to 1.44; P for trend=0.013) among men and ready-to-eat/heat mixed dishes among women (1.17, 1.01 to 1.36; P for trend=0.02) was associated with increased risk of colorectal cancer; yogurt and dairy based desserts were negatively associated with the risk of colorectal cancer among women (hazard ratio 0.83, 0.71 to 0.97; P for trend=0.002).

**Conclusions:**

In the three large prospective cohorts, high consumption of total ultra-processed foods in men and certain subgroups of ultra-processed foods in men and women was associated with an increased risk of colorectal cancer. Further studies are needed to better understand the potential attributes of ultra-processed foods that contribute to colorectal carcinogenesis.

## Introduction

Colorectal cancer is the third most commonly diagnosed malignancy among both men and women in the United States and the second leading cause of death from cancer worldwide.^1^ ^2^ Diet has been recognized as an important modifiable risk factor for colorectal cancer.^3^ Meanwhile, ultra-processed foods (that is, industrial ready-to-eat or ready-to-heat formulations made of little or no whole foods) now contribute 57% of total daily calories consumed by American adults, which has been continuously increasing in the past two decades.^4^ These foods are usually high in added sugar, oils/fats, and refined starch, altering gut microbiota composition unfavorably^5^ and contributing to increased risk of weight gain and obesity, an established risk factor for colorectal cancer. Diets high in ultra-processed foods are also usually low in nutrients and bioactive compounds that are beneficial for the prevention of colorectal cancer, such as fiber, calcium, and vitamin D.^6^ ^7^ ^8^ ^9^ Beyond poor nutrition profiles, ultra-processed foods commonly contain food additives such as dietary emulsifiers and artificial sweeteners, some types of which have been suggested to increase the pro-inflammatory potential of the gut microbiome,^10^ ^11^ ^12^ ^13^ promoting colon carcinogenesis.^11^ ^13^ Furthermore, potential carcinogens may also be formed during the processing of meats containing sodium nitrates (for example, nitrosamines)^14^ or heat treatment (for example, acrylamide)^15^ or may migrate from the packaging of ultra-processed foods (for example, bisphenol A).^16^

In a multicentric, population based case-control study in Spain, Romaguera and colleagues reported a positive association between ultra-processed food consumption and the risk of colorectal cancer.^17^ Only one prospective cohort study has evaluated the association between ultra-processed food consumption and risk of cancer,^18^ which reported that high ultra-processed food consumption was associated with an increased risk of developing all cancers but not of colorectal cancer among participants of the French NutriNet-Sante Cohort.^18^ The null findings for colorectal cancer might be explained, at least in part, by a relatively small number of participants who developed colorectal cancer during a limited length of follow-up. Colorectal cancer is also considered a heterogeneous disease, with potentially distinct etiology for tumors developed at different anatomic sites—namely, the proximal colon, distal colon, and rectum.^19^ Therefore, we aimed to evaluate the association between ultra-processed food consumption and risk of colorectal cancer overall and by anatomic subsites among men and women who participated in three large prospective cohort studies.

## Methods

We used data from three large prospective cohorts in the US. The Nurses’ Health Study (NHS) included 121 700 registered female nurses aged 30 to 55 years at baseline in 1976,^20^ the Nurses’ Health Study II (NHS-II) enrolled 116 429 female nurses aged 25 to 42 years at baseline in 1989,^20^ and the Health Professionals Follow-up Study (HPFS) enrolled 51 529 male health professionals aged 40 to 75 years at baseline in 1986.^21^ At baseline and every two years thereafter, study participants were mailed a questionnaire collecting information on demographics, lifestyle, and medical conditions. The average follow-up rate was greater than 90% in all three cohorts.

In this analysis, we used 1986 for the NHS (n=102 267), 1991 for the NHS II (n=108 453), and 1986 for the HPFS as the baseline, since when the Food Frequency Questionnaires (FFQs) were the most complete and comparable across studies.^20^ ^22^ We excluded participants with previously diagnosed cancer (except for non-melanoma skin cancer), with a history of ulcerative colitis, with implausibly high or low caloric intakes (<800 or >4200 kcal/d for men; <600 or >3500 kcal/d for women), and with a high number of blank items on their FFQs (>70) at baseline (flowchart shown in supplementary figure A). This resulted in the inclusion of 67 425 women from the NHS, 92 482 women in NHS II, and 46 341 men from the HPFS for analysis.

### Assessment of ultra-processed food consumption

Dietary intake was assessed by validated semiquantitative FFQs with about 130 food items, administered every four years since 1986 in NHS and HPFS and 1991 in NHS II.^23^ ^24^ The FFQs continue to be updated to capture new and reformulated products.^25^ The NOVA classification categorizes foods into four groups^26^: unprocessed or minimally processed foods, processed culinary ingredients, processed foods, and ultra-processed foods. Examples of ultra-processed foods include carbonated drinks, sausages, biscuits, candies, instant soups/noodles, sweet/savory packaged snacks, and sugary milk based and fruit based drinks. Three researchers (NK, SLR, EMS) worked independently to assign each food item to a NOVA group.^27^ ^28^ The three researchers reached a consensus on more 70% of all food items at the first attempt of the classification. When discordance existed in classifying a food item, we used discussions with an expert group and additional resources (research dieticians, cohort specific documents, online grocery store scans) to guide the final categorization. For nine food items that lacked sufficient details in the resource documents to support their classification (for example, “popcorn,” “soy milk,” “pancakes or waffles,” “pie, home-baked or ready-made,” “beef, pork, lamb sandwich,” “tomato sauce”), we adopted a conservative approach by assigning these items as non-ultra-processed in the primary categorization and as ultra-processed foods in a sensitivity analysis. Although distilled alcohols meet the definition for ultra-processed foods, we removed this item from the summary of overall ultra-processed food intake to avoid mixing the effect of alcohol with that of ultra-processed food. Given that the carcinogenic effect of ultra-processed foods may go beyond the calories they represent, we estimated ultra-processed food intake as servings per day, with energy adjustments made using the residual methods.^29^

We further estimated the percentage of total energy from ultra-processed foods for each participant as the exposure variable in a sensitivity analysis. To further investigate whether the association between overall ultra-processed food consumption and colorectal cancer was driven by specific food groups, we further categorized ultra-processed foods into mutually exclusive subgroups (supplementary table A).^30^

### Ascertainment of colorectal cancer cases

On each biennial follow-up questionnaire, participants were asked to report any cancer diagnosis in the previous two years. After receiving permission from the study participants, study physicians blinded to exposure data reviewed the medical records and pathological reports to confirm the diagnosis and extract information on anatomic location. We used information from various sources, including next of kin, the National Death Index, death certificates, and medical records, to confirm the diagnosis in participants who died from colorectal cancer but had not reported a diagnosis on a questionnaire.^31^ We defined proximal cancers as those that occurred in the cecum, ascending colon, and transverse colon; distal colon cancers as those in the descending and sigmoid colon; and rectal cancers as those in the rectosigmoid junction and rectum.

### Assessment of covariates

Self-administered questionnaires were sent to participants biennially to assess medical and lifestyle factors, including smoking, physical activity, alcohol intake, endoscopy status, regular use of aspirin and other non-steroidal anti-inflammatory drugs, family history of colorectal cancer, weight, and height, as well as menopausal status and postmenopausal hormone use for women, in all three cohorts as previously described.^20^ ^22^ ^32^ On the basis of dietary data assessed by FFQ, we calculated the “western” dietary pattern score derived from principal component analyses and the Alternative Healthy Eating Index-2010 score (AHEI-2010),^33^ ^34^ both as previously described. The percentage of missing values for each covariate was less than 1% (supplementary table B).

### Statistical analysis

We calculated person years of follow-up from the return date of the first FFQ until the date of death, diagnosis of colorectal cancer, or end of follow-up (1 June 2014 for NHS, 1 June 2015 for NHS II, and 31 January 2014 for HPFS), whichever came first. To better represent long term habitual intake and minimize random measurement errors, we calculated the cumulative average of energy adjusted servings of ultra-processed food intake per day and then categorized them into fifths. For example, in the NHS, we used the cumulative average of ultra-processed food intake from 1986 and 1990 as the exposure for the follow-up period from 1990 to 1994 and the cumulative average from 1986, 1990, and 1994 for the follow-up period between 1994 and 1996. Owing to the high within individual correlations in dietary intake between adjacent data cycles, we carried forward non-missing dietary intake data from the previous data cycle to replace missing data in the next cycle. We treated covariates similarly.

We used Cox proportional hazards regression models to estimate the hazard ratios and 95% confidence intervals for risk of colorectal cancer in association with ultra-processed food intake. To account for repeated measures of food intake and covariates over time, we constructed the model on the basis of a counting process data structure for handling time varying covariates.^35^ ^36^ All analyses were stratified simultaneously by age (in years) and calendar year of return of questionnaire (every two years since the baseline questionnaire), allowing for the finest possible control of confounding for age and secular trends.^36^ We tested departure from the proportional hazards assumption by using likelihood ratio tests comparing models with and without the interaction terms of calendar time by exposure. We found no evidence of departure from the proportional hazard assumption (all P values interaction >0.05).

In the multivariable model, we adjusted for potential confounding factors, including race (white/non-white), family history of cancer (yes or no), history of endoscopy (yes or no), physical activity (in metabolic equivalent-hours/week: <3, 3-8.9, 9-17.9, 18-26.9, or ≥27), smoking status and pack years of smoking (never, past smoker with pack years <5, past smoker with pack years ≥5, current smoker with pack years <20, current smoker with pack years ≥20), total alcohol intake (in g/day: <5, 5-9.9, 10-14.9, 15-29.9, or ≥30), total caloric intake (in fifths), and regular aspirin use (yes or no) and additionally for menopausal status (yes or no) and post-menopausal hormone use (yes or no) in women.

On the basis of previous findings on the different pattern of the association of dietary factors with colorectal cancer risk between the sexes, we did the analyses among men (in HPFS) and women (in pooled data from NHS and NHS II) separately. We used the likelihood ratio test to examine the heterogeneity of risk estimates by sex in pooled data of the three cohorts. We examined the potential non-linear relation between ultra-processed food consumption and risk of colorectal cancer by using the restricted cubic spline analysis.^37^ We examined the statistical significance for non-linearity with the likelihood ratio test, comparing the model with only the linear term against the model with both the linear and the cubic spline terms. None of the P values for non-linearity reached statistical significance (supplementary table C).

As body mass index is a possible intermediate step linking ultra-processed food consumption with risk of colorectal cancer, we additionally adjusted for body mass index (model S1) in the multivariable model as a sensitivity analysis. Similarly, we adjusted for variables that could serve as both confounders and mediators, including western dietary pattern score (model S2),^33^ the AHEI-2010 score (model S3), or intakes of processed meats, fruits, vegetables, whole grains, calcium, vitamin D, and folate (model S4)^3^ in separate multivariable models in sensitivity analyses. A causal diagram for the mechanism of the adjusted covariates in the main analysis and additional models in sensitivity analysis are shown in Supplemental figure B. Sensitivity analyses also assessed use of the alternative classification of food items with insufficient information on processing method, use of percentage of total energy from ultra-processed foods as the exposure variable, and exclusion of participants with more than 10 missing items in the FFQs at baseline.

To further overcome the potential collider stratification bias due to the adjustment of time varying covariates in the Cox models, we emulated a target trial and applied the parametric g-formula to estimate the effect of a hypothetical intervention of limiting ultra-processed food consumption on risk of colorectal cancer.^38^ ^39^ The detailed target trial specifications are provided in the supplementary methods.

The association between ultra-processed food consumption and risk of colorectal cancer may be modified by genetic predisposition and other lifestyle factors such as physical activity, smoking, and body weight.^40^ We therefore further evaluated the potential effect modification by family history of colorectal cancer, body mass index, physical activity, smoking, and AHEI-2010 score on the association of ultra-processed food and colorectal cancer by doing stratified analysis. We used the likelihood ratio test to examine the potential effect modification by comparing models with and without the interaction term of fifths of ultra-processed food intake and the indicator variables for categories of potential effect modifiers.

We used SAS software, version 9.4 for UNIX, for all analyses. We set statistical significance at a two sided P value <0.05.

### Patient and public involvement

This study used data from three large prospective cohort studies that were not specifically designed for the current aim. Therefore, we were not able to involve patients in setting the research question or the outcome measures, or in the design and implementation of the study. We plan to disseminate these findings to participants in our annual newsletter and the general public in a press release.

## Results

**Fig 1:**
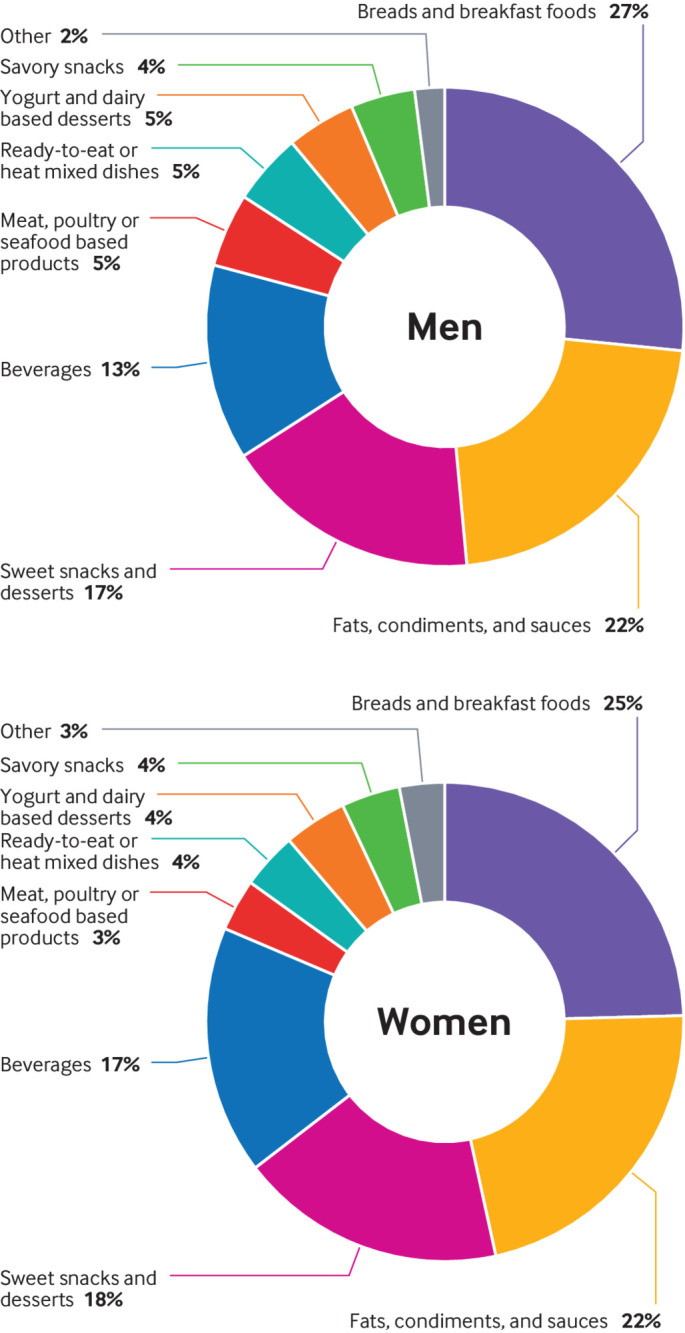
Relative contribution (%) of each food group to energy adjusted servings per day of ultra-processed food consumption among men and women. Ultra-processed bread and breakfast foods include breakfast bars, cold breakfast cereal, English muffins, bagels, rolls, rye, pumpernickel bread, white bread, and whole grain bread. Meat/poultry/seafood based ready-to-eat products include bacon/ beef/pork hotdogs, chicken/turkey hotdogs, salami, bologna, processed meat sandwiches, processed meats, sausages, and breaded fish cakes/pieces/sticks. Packaged sweet snacks and desserts include readymade brownies, cake, cookies, doughnuts, pies, muffins or biscuits, sweet roll, coffee cake, candy bars, chocolate bars, energy bars, high protein and low carbohydrate candy bars, apple sauce, jams, jellies, preserves, and honey. Fats, condiments, and sauces include ketchup, red chili sauce, salad dressings, mayonnaise (regular and low fat), salsa, margarine, spread butter, soy sauce, non-dairy coffee whitener, and cream cheese. Sugar or artificially sweetened beverages include caffeine-free Coke, Coke/Pepsi/Cola, dairy coffee drink, Hawaiian punch, low calorie soda, caffeine-free low calorie soda, Pepsi, 7-up, other carbonated beverages, and other low calorie Cola with caffeine. Yogurt and dairy based desserts include frozen yogurt, sherbet, ice cream, artificially sweetened yogurt, and flavored yogurt. Ready-to-eat/heat mixed dishes include pizza, chowder or cream soup, soup made with bouillon, readymade soup from cans, and French fries. Packaged savory snacks include regular crackers, fat-free light crackers, and fat-free popcorn. Other ultra-processed foods include Nutrasweet or equivalent, other artificial sweeteners, and Splenda

Over the 24 to 28 years of follow-up, we documented 1294 cases of colorectal cancer among men in HPFS and 1922 cases among women in NHS and NHS II. Among both men and women, compared with those in the lowest fifth of ultra-processed food intake, participants in the highest fifth were more likely to be current smokers or smoke more pack years; to have a higher body mass index, lower consumption of alcohol, and a lower level of physical activity; and to consume a lower amount of dietary fiber, folate, calcium, vitamin D, and whole grains and a higher intake of fat, added sugars, and processed meats (table 1). They were also more likely to use aspirin regularly and to have a lower overall dietary quality score measured by AHEI-2010. The energy adjusted mean of ultra-processed food consumption was 6.5 (SD 2.3) servings/day for women and 6.6 (2.3) servings/day for men. Among both men and women, subgroups contributing the largest share of ultra-processed food consumption were ultra-processed bread and breakfast foods (1.7 (1.2) servings/day among men, 1.6 (1.1) servings/day among women); fats, condiments, and sauces (1.4 (1.1) servings/day among men, 1.4 (1.1) servings/day among women); and packaged sweet snacks and desserts (1.1 (0.9) servings/day among men, 1.2 (0.9) servings/day among women) (fig 1).

**Table 1:** Baseline characteristics of study participants by fifths of ultra-processed food consumption among men and women in three large cohorts*. Values are means (standard deviations) unless stated otherwise†

Characteristics	Energy adjusted servings ultra-processed food intake per day						
	Men (HPFS; n=46 341)				Women (NHS+NHS II; n=155 907)		
	Fifth 1 (n=9259)	Fifth 3 (n=9277)	Fifth 5 (n=9245)		Fifth 1 (n=13 395)	Fifth 3 (n=13 548)	Fifth 5 (n=13 480)
Median (range) ultra-processed food intake, energy adjusted servings/day*	3.3 (0.1-4.1)	5.8 (5.2-6.3)	9.3 (7.9-27.1).		3.3 (0.1-4.0)	5.4 (5.0-5.9)	8.4 (7.2-26.7)
Age, years	54.9 (9.7)	53.5 (9.7)	53.7 (9.7)		53.0 (6.9)	51.97 (7.2)	52.9 (7.3)
No (%) white	9271 (89.3)	8432 (90.9)	8501 (91.9)		12 918 (96.5)	13 288 (98.1)	13 258 (98.3)
No (%) family history of colorectal cancer	1381 (14.7)	1328 (14.4)	1361 (14.8)		2651 (19.7)	2622 (19.5)	2690 (19.9)
Body mass index	25.1 (3.3)	25.5 (3.2)	25.8 (3.5)		24.3 (4.5)	25.0 (4.9)	25.4 (5.3)
No (%) past smoker	3762 (41.6)	3812 (43.0)	4107 (46.3)		4804 (35.8)	4706 (34.9)	4476 (33.2)
No (%) current smoker	802 (9.1)	881 (9.9)	1085 (12.2)		2647 (19.8)	2726 (20.1)	3429 (25.6)
Pack years of smoking	11.7 (17.5)	13.0 (18.4)	16.0 (20.5)		11.9 (17.5)	12.1 (17.6)	15.0 (19.9)
Alcohol, g/day	13.7 (17.9)	11.1 (14.7)	9.1 (13.6)		8.2 (12.3)	6.4 (10.0)	5.0 (9.1)
Physical activity, MET-hours/week	23.3 (26.7)	20.2 (24.3)	18.6 (23.5)		16.8 (22.3)	14.3 (21.3)	11.2 (18.6)
No (%) history of endoscopy	733 (7.8)	773 (8.4)	780 (8.5)		446 (3.3)	435 (3.3)	451 (3.3)
No (%) regular aspirin use (≥2 tablets/week)	2466 (26.1)	2756 (30.0)	29 220 (31.7)		6045 (44.8)	6560 (48.7)	6763 (50.0)
No (%) postmenopausal hormone use	NA	NA	NA		4276 (30.7)	4106 (31.5)	4309 (31.2)
No (%) postmenopausal status	NA	NA	NA		9319 (67.0)	8712 (66.5)	9301 (67.6)
Dietary intake:							
Total energy intake, kcal/d	1879 (569)	2007 (604)	1985 (613)		1713 (528)	1794 (529)	1745 (524)
Dietary fiber, g/day	23.4 (8.3)	20.7 (6.3)	19.4 (6.6)		19.5 (6.2)	17.6 (5.0)	15.9 (4.9)
Folate intake, μg/day	537 (319)	473 (268)	445 (259)		453 (247)	405 (212)	362 (213)
Total calcium, mg/day	962 (508)	887 (402)	867 (398.7)		1173 (551)	1079 (491)	1007 (493)
Total vitamin D, IU/day	464 (365)	399 (300)	374 (281)		395 (288)	335 (241)	309 (238)
Total fat, mg/day	66.2 (15.8)	71.9 (12.9)	75.0 (13.4)		54.4 (10.9)	58.1 (9.4)	61.1 (9.9)
Added sugars, g/day	33 (22.3)	49.4 (30.7)	56.6 (39.0)		32.1 (21.4)	42.4 (25.8)	47.0 (31.1)
Processed meats, servings/day	0.2 (0.3)	0.4 (0.4)	0.5 (0.6)		0.19 (0.21)	0.31 (0.3)	0.36 (0.4)
Red meats, servings/day	0.5 (0.5)	0.6 (0.5)	0.6 (0.4)		0.72 (0.6)	0.78 (0.51)	0.71 (0.47)
Whole grains, g/day	24.8 (23.4)	21.3 (18)	21.4 (20.7)		16.6 (16.2)	14.0 (12.8)	13.4 (13.3)
Dairy, servings/day	2.2 (1.8)	2.3 (1.6)	2.1 (1.5)		2.7 (1.8)	2.6 (1.6)	2.2 (1.5)
AHEI-2010 score	51.7 (11.3)	46.4 (10.3)	43.9 (10.3)		56.9 (11.9)	52.1 (10.8)	48.6 (10.6)

AHEI=Alternative Health Eating Index; HPFS=Health Professionals Follow-up Study; MET=metabolic equivalent tasks; NA=not applicable; NHS=Nurses’ Health Study.

* Energy adjusted intake=a+b, where a=residual for participant from regression model with intake of ultra-processed food as dependent variable and total caloric intake as independent variable and b=expected ultra-processed food intake for person with mean caloric intake (2000 kcal/d for HPFS participants; 1600 kcal/d for NHS participants; 1800 kcal/d for NHS II participants).

† Values other than age are standardized to age distribution of study population.

After multivariable adjustments, men who consumed ultra-processed foods in the highest fifth had a 29% higher risk of developing colorectal cancer than did those in the lowest fifth (hazard ratio 1.29, 95% confidence interval 1.08 to 1.53; P for trend=0.01) (table 2). However, we found no significant association among women (hazard ratio for highest versus lowest fifth 1.04, 0.90 to 1.20; P for trend=0.29). When evaluating the risk for anatomic subsites of colorectal cancer separately, we found that high ultra-processed food consumption was associated with a 72% higher risk of distal colon cancer in men (hazard ratio 1.72, 1.24 to 2.37; P for trend<0.001) but found no significant dose-response association for proximal colon cancer or rectal cancer. Ultra-processed food consumption was not significantly associated with the risk of anatomic subsite of colorectal cancer among women.

**Table 2:** Colorectal cancer risk by fifths of ultra-processed food consumption among men and women. Values are hazard ratios (95% confidence intervals) unless stated otherwise

	Energy adjusted servings per day of ultra-processed food intake*							P for heterogeneity between sexes‡
	Fifths					P for trend†	Continuous	
	1	2	3	4	5			
**Colorectal cancer**								
Men (HPFS):								0.045
Cases/person years	234/205 781	276/219 114	235/221 181	263/218 770	286/209 632	-	-	
Age adjusted model	Reference	1.19 (1.00 to 1.42)	1.00 (0.83 to 1.19)	1.13 (0.95 to 1.35)	1.24 (1.04 to 1.47)	0.04	1.03 (1.00 to 1.06)	
Multivariable adjusted model§	Reference	1.22 (1.02 to 1.45)	1.04 (0.86 to 1.25)	1.17 (0.98 to 1.40)	1.29 (1.08 to 1.53)	0.01	1.04 (1.01 to 1.06)	
Women (NHS+NHS II):								
Cases/person years	379/823 070	360/869668	385/873557	396/861866	402/824317	-	-	
Age adjusted model	Reference	0.91 (0.78 to 1.05)	0.96 (0.84 to 1.11)	1.01 (0.87 to 1.16)	1.08 (0.94 to 1.24)	0.08	1.02 (1.00 to 1.05)	
Multivariable adjusted model§	Reference	0.90 (0.78 to 1.04)	0.95 (0.83 to 1.10)	0.99 (0.85 to 1.14)	1.04 (0.90 to 1.20)	0.29	1.01 (0.98 to 1.04)	
**Proximal colon cancer**								
Men (HPFS):								0.31
Cases	72	107	74	97	93	-	-	
Age adjusted model	Reference	1.51 (1.12 to 2.03)	1.02 (0.74 to 1.42)	1.36 (1.00 to 1.85)	1.32 (0.97 to 1.79)	0.22	1.03 (0.98 to 1.08)	
Multivariable adjusted model§	Reference	1.52 (1.12 to 2.05)	1.04 (0.75 to 1.43)	1.38 (1.01 to 1.87)	1.34 (0.98 to 1.82)	0.20	1.03 (0.98 to 1.08)	
Women (NHS+NHS II):								
Cases	151	148	168	158	172	-	-	
Age adjusted model	Reference	0.93 (0.74 to 1.17)	1.06 (0.85 to 1.31)	1.00 (0.80 to 1.25)	1.16 (0.93 to 1.45)	0.11	1.03 (0.99 to 1.07)	
Multivariable adjusted model§	Reference	0.92 (0.73 to 1.15)	1.04 (0.83 to 1.30)	0.98 (0.78 to 1.23)	1.11 (0.89 to 1.39)	0.24	1.02 (0.98 to 1.06)	
**Distal colon cancer**								
Men (HPFS):								0.001
Cases	62	68	69	69	100	-	-	
Age adjusted model	Reference	1.11 (0.79 to 1.57)	1.11 (0.79 to 1.57)	1.13 (0.80 to 1.59)	1.62 (1.18 to 2.23)	0.002	1.08 (1.03 to 1.14)	
Multivariable adjusted model§	Reference	1.15 (0.81 to 1.62)	1.17 (0.83 to 1.65)	1.17 (0.83 to 1.66)	1.72 (1.24 to 2.37)	<0.001	1.09 (1.04 to 1.15)	
Women (NHS+NHS II):								
Cases	91	72	93	101	98			
Age adjusted model	Reference	0.76 (0.56 to 1.03)	0.98 (0.73 to 1.30)	1.07 (0.81 to 1.42)	1.10 (0.82 to 1.46)	0.13	1.04 (0.99 to 1.10)	
Multivariable adjusted model§	Reference	0.77 (0.56 to 1.04)	0.97 (0.73 to 1.30)	1.06 (0.79 to 1.41)	1.07 (0.80 to 1.43)	0.19	1.04 (0.98 to 1.09)	
**Rectal cancer**								
Men (HPFS):								0.75
Cases	51	59	56	50	51	-	-	
Age adjusted model	Reference	1.15 (0.79 to 1.68)	1.07 (0.73 to 1.57)	0.97 (0.65 to 1.43)	1.01 (0.68 to 1.48)	0.72	0.99 (0.93 to 1.05)	
Women (NHS+NHS II):								
Cases	77	89	71	78	83	-	-	
Age adjusted model	Reference	1.10 (0.81 to 1.49)	0.88 (0.63 to 1.21)	0.98 (0.71 to 1.34)	1.10 (0.81 to 1.51)	0.70	1.01 (0.96 to 1.07)	
Multivariable adjusted model§	Reference	1.11 (0.82 to 1.51)	0.88 (0.63 to 1.21)	0.96 (0.70 to 1.33)	1.08 (0.79 to 1.49)	0.84	1.00 (0.95 to 1.06)	

HPFS=Health Professionals Follow-up Study; NHS=Nurses’ Health Study.

* Energy adjusted intake=a+b, where a=residual for participant from regression model with intake of ultra-processed food as dependent variable and total caloric intake as independent variable and b=expected ultra-processed food intake for person with mean caloric intake (2000 kcal/d for HPFS participants; 1600 kcal/d for NHS participants; 1800 kcal/d for NHS II participants).

† P value for linear trend of colorectal cancer risk across fifths of ultra-processed food consumption was obtained by assigning fifth medians to each participant in fifth as ordinal variable, adjusted for same set of covariates as below§.

‡ P for heterogeneity between sexes was examined using likelihood ratio test comparing multivariable adjusted model with and without interaction term of sex and exposure in pooled dataset of three cohorts.

§ Multivariable adjusted model was adjusted for age, calendar year of current questionnaire, race, family history of cancer, history of endoscopy, total alcohol intake (in g/day: <5, 5-9.9, 10-14.9, 15-29.9, or ≥30), physical activity (in metabolic equivalent-hours/week: <3, 3–8.9, 9–17.9, 18–26.9, or ≥27), smoking status and pack years of smoking (never, past smoker with pack years <5, past smoker with pack years ≥5, current smoker with pack years <20, current smoker with pack years ≥20), total caloric intake (fifths), and regular aspirin use and additionally for menopausal status and postmenopausal hormone use in women. Results from NHS and NHS II were pooled and summary estimates generated using random effect models.

Sensitivity analyses showed similar results after further adjustment for body mass index (model S1) (supplementary table D), western dietary pattern score (model S2), or the AHEI-2010 score (model S3). Adjustment for intake of foods and nutrients including processed meats, fruits, vegetables, whole grains, calcium, vitamin D, and folate (model S4) attenuated the association of ultra-processed food consumption with overall colorectal cancer risk among men to non-significant (hazard ratio for highest versus lowest fifth 1.18, 0.98 to 1.42; P for trend=0.15), whereas the association with distal colon cancer remained significant (1.53, 1.09 to 2.15; P for trend=0.01). In addition, the results were not materially altered after recategorization of the undetermined food items as ultra-processed foods (supplementary table E), use of percentage of total energy of ultra-processed foods as the exposure variable (supplementary table F), or exclusion of participants with more than 10 missing items in the FFQs at baseline (supplementary table G) in sensitivity analyses.

In the emulated targeted trial analysis, compared with no intervention (on average 6.6 servings per day of consumption), the dietary intervention scenario of restricting ultra-processed food consumption to four servings per day was associated with a risk ratio of 0.94 (95% confidence interval 0.89 to 1.00) for developing colorectal cancer and 0.82 (0.72 to 0.94) for developing distal colon cancer among men (supplementary table H). We found no association among women. We did not observe any significant interactions between potential effect modifiers and ultra-processed food consumption for colorectal cancer risk (all P for heterogeneity>0.05) (supplementary table I).

Among subgroups of ultra-processed foods (table 3), we observed a positive association with colorectal cancer for higher consumption of meat/poultry/seafood based ready-to-eat products (hazard ratio for highest versus lowest fifth 1.44, 1.20 to 1.73; P for trend<0.001) and sugar sweetened beverages (1.21, 1.01 to 1.44; P for trend=0.01) among men and for higher consumption of ready-to-eat/heat mixed dishes among women (1.17, 1.01 to 1.36; P for trend=0.02). In addition, the consumption of yogurt and dairy based desserts was negatively associated with the risk of colorectal cancer among women (hazard ratio 0.83, 0.71 to 0.97; P for trend=0.002).

**Table 3:** Multivariable adjusted associations of ultra-processed food subgroups with colorectal cancer risk among men and women. Values are hazard ratios (95% confidence intervals) unless stated otherwise

Subgroup	Energy adjusted servings of subgroup intake*						
	Fifths†					P for trend‡	Continuous
	1	2	3	4	5		
Ultra-processed bread and breakfast food:							
Men	Reference	1.03 (0.86 to 1.23)	1.18 (0.99 to 1.41)	1.05 (0.88 to 1.26)	1.06 (0.88 to 1.27)	0.66	1.02 (0.95 to 1.09)
Women	Reference	1.09 (0.94 to 1.27)	1.03 (0.89 to 1.19)	0.92 (0.79 to 1.07)	1.00 (0.86 to 1.16)	0.37	0.97 (0.91 to 1.04)
Fat, condiments, and sauces:							
Men	Reference	1.14 (0.95 to 1.36)	0.99 (0.82 to 1.19)	1.22 (1.03 to 1.46)	1.14 (0.95 to 1.36)	0.13	1.05 (0.98 to 1.12)
Women	Reference	0.90 (0.78 to 1.04)	0.93 (0.80 to 1.07)	0.99 (0.86 to 1.14)	0.97 (0.84 to 1.12)	0.81	1.01 (0.95 to 1.07)
Packaged sweet snacks and desserts:							
Men	Reference	0.95 (0.80 to 1.14)	1.03 (0.86 to 1.22)	1.02 (0.86 to 1.22)	1.06 (0.90 to 1.26)	0.31	1.05 (0.96 to 1.14)
Women	Reference	1.04 (0.90 to 1.20)	1.04 (0.90 to 1.21)	1.05 (0.91 to 1.22)	0.98 (0.85 to 1.14)	0.70	0.98 (0.90 to 1.07)
Sugar sweetened beverages:							
Men	Reference	0.96 (0.81 to 1.14)	1.10 (0.93 to 1.30)	1.10 (0.93 to 1.32)	1.21 (1.01 to 1.44)	0.013	1.28 (1.05 to 1.55)
Women	Reference	0.93 (0.80 to 1.07)	1.01 (0.88 to 1.17)	1.09 (0.94 to 1.26)	1.04 (0.89 to 1.21)	0.26	1.09 (0.94 to 1.25)
Artificially sweetened beverages:							
Men	Reference	1.06 (0.88 to 1.27)	1.15 (0.97 to 1.37)	1.11 (0.93 to 1.32)	1.14 (0.95 to 1.38)	0.31	1.06 (0.95 to 1.19)
Women	Reference	1.06 (0.92 to 1.23)	1.08 (0.93 to 1.25)	1.01 (0.87 to 1.18)	1.07 (0.91 to 1.25)	0.78	1.01 (0.95 to 1.07)
Ready-to-eat/heat mixed dishes:							
Men	Reference	1.00 (0.85 to 1.19)	1.11 (0.94 to 1.31)	1.22 (1.03 to 1.44)	0.98 (0.82 to 1.18)	0.66	1.08 (0.76 to 1.53)
Women	Reference	1.06 (0.91 to 1.22)	1.03 (0.89 to 1.19)	1.15 (0.99 to 1.33)	1.17 (1.01 to 1.36)	0.02	1.66 (1.09 to 2.54)
Meat/poultry/seafood based ready-to-eat products:							
Men	Reference	1.20 (0.99 to 1.44)	1.29 (1.07 to 1.55)	1.51 (1.26 to 1.81)	1.44 (1.20 to 1.73)	<.0001	1.63 (1.29 to 2.05)
Women	Reference	1.10 (0.95 to 1.27)	1.10 (0.95 to 1.28)	1.20 (1.03 to 1.38)	1.14 (0.98 to 1.32)	0.08	1.30 (0.97 to 1.73)
Packaged savory snacks:							
Men	Reference	1.13 (0.95 to 1.36)	1.07 (0.90 to 1.29)	1.09 (0.91 to 1.31)	1.07 (0.90 to 1.27)	0.90	1.02 (0.79 to 1.31)
Women	Reference	0.92 (0.79 to 1.06)	0.98 (0.85 to 1.13)	0.87 (0.75 to 1.00)	0.89 (0.78 to 1.03)	0.13	0.82 (0.63,1.06)
Yogurt and dairy based desserts:							
Men	Reference	1.17 (0.98 to 1.40)	1.13 (0.95 to 1.35)	1.09 (0.92 to 1.31)	1.11 (0.93 to 1.33)	0.66	1.07 (0.78 to 1.48)
Women	Reference	1.02 (0.89 to 1.18)	1.10 (0.96 to 1.27)	0.98 (0.85 to 1.13)	0.83 (0.71 to 0.97)	0.002	0.66 (0.50 to 0.86)
Other ultra-processed foods:							
Men	Reference	0.91 (0.71 to 1.15)	1.14 (0.94 to 1.39)	1.17 (0.96 to 1.41)	1.18 (0.98 to 1.43)	0.18	1.22 (0.91 to 1.64)
Women	Reference	1.11 (0.94 to 1.31)	1.20 (1.01 to 1.42)	1.10 (0.94 to 1.30)	1.08 (0.92 to 1.28)	0.73	0.97 (0.80 to 1.16)

* Energy adjusted intake=a+b, where a=residual for participant from regression model with intake of ultra-processed food as dependent variable and total caloric intake as independent variable and b=expected ultra-processed food intake for person with mean caloric intake (2000 kcal/d for HPFS participants; 1600 kcal/d for NHS participants; 1800 kcal/d for NHS II participants).

† Multivariable adjusted model was adjusted for age, calendar year of current questionnaire, race, family history of cancer, history of endoscopy, total alcohol intake (in g/day: <5, 5-9.9, 10-14.9, 15-29.9, or ≥30), physical activity (in metabolic equivalent-hours/week: <3, 3–8.9, 9–17.9, 18–26.9, or ≥27), smoking status and pack years of smoking (never, past smoker with pack years <5, past smoker with pack years ≥5, current smoker with pack years <20, current smoker with pack years ≥20), total caloric intake (fifths), and regular aspirin use and additionally for menopausal status and postmenopausal hormone use in women.

‡ P value for linear trend of colorectal cancer risk across fifths of ultra-processed food subgroup consumption was obtained by assigning fifth medians to each participant in fifth as an ordinal variable, adjusted for same set of covariates as above†.

## Discussion

In this analysis of three large prospective cohorts with nearly three decades of follow-up, we found a positive association between consumption of ultra-processed food and risk of colorectal cancer among men, and the association was limited to distal colon cancer. Among subgroups of ultra-processed foods, we observed a positive association with colorectal cancer for higher consumption of meat/poultry/seafood based ready-to-eat products and sugar sweetened beverages among men and higher consumption of ready-to-eat/heat mixed dishes among women; we observed a negative association with colorectal cancer for yogurt and dairy based desserts among women.

### Comparison with other studies

This study is among the first that has detected a positive association between ultra-processed food consumption and risk of colorectal cancer among men. Consumption of ultra-processed foods may contribute to poorer overall dietary quality,^7^ ^8^ ^9^ as well as increased risk of weight gain and obesity,^41^ ^42^ which is an established risk factor for colorectal cancer.^43^ ^44^ However, our results show that the association between ultra-processed food consumption and colorectal cancer among men was largely independent of body mass index. Also, ultra-processed food was associated with risk of distal colon cancer independently of different dietary indices including the western dietary pattern score, AHEI score, and specific food groups and nutrients that have been associated with colorectal cancer risk. Thus, additional attributes of ultra-processed foods beyond dietary quality may be involved in colorectal carcinogenesis. For example, ultra-processed foods commonly contain food additives such as emulsifiers and artificial sweeteners, which may alter gut microbiota, promoting inflammation and colon carcinogenesis.^11^ ^12^ ^13^ ^45^ ^46^ ^47^ In addition to additives, newly formed contaminants with carcinogenesis potentials (for example, acrylamide) are found in various ultra-processed products that have undergone heat treatment, especially French fries.^48^ ^49^ ^50^ ^51^ ^52^ Ultra-processed foods may also contain contaminants that migrate from plastic packaging, such as bisphenol A, which the European Chemicals Agency judges to be “a substance of very high concern.” Further studies are needed to investigate the different potential carcinogenic pathways of ultra-processed foods.

Why the association was seen in men but not in women is unclear. A previous review study found that the associations of dietary patterns with colorectal cancer were more consistently significant and stronger in men than women.^53^ Using the same data source as in this study, Petimar and colleagues found that the adherence to the Dietary Approaches to Stop Hypertension (DASH), Alternative Mediterranean Diet (AMED), and AHEI-2010 was associated with lower risk of colorectal cancer among men but not among women.^34^ Potential explanations for such differing sex patterns may involve the effect of obesity and sex hormones. For men and postmenopausal women, estrogen is mainly produced in fat tissues.^54^ In women, a high estrogen to testosterone ratio may decrease the risk of colorectal cancer, whereas it may increase the risk of colorectal cancer in men.^54^ In addition, ultra-processed foods include a variety of foods, with some products being healthier than others. Different food choices made within ultra-processed foods could contribute to differential associations with health outcomes. For example, relatively healthier food choices may have been made within the category of “yogurt and dairy based desserts” among women, and thus the protective effects (for example, due to higher calcium contents) may overweigh the harmful effects (for example, due to higher sugar contents). Despite the lack of significant association between overall ultra-processed food intake and colorectal cancer risk in women, this study showed that consumption of ready-to-eat/heat mixed dishes was associated with an increased risk of colorectal cancer. Such findings support the recommendation by the World Cancer Research Fund International/American Institute for Cancer Research to limit the intake of “fast foods” for the primary prevention of cancer.

The differing role of diet on colorectal cancer risks at specific anatomic subsites remains unclear. Stronger associations for overall dietary patterns,^33^ ^34^ ^53^ processed meat consumption,^55^ and dietary calcium intake^56^ with risk of distal colon cancer than colorectal cancer at other sites had been seen in previous studies, which are in accordance with the findings of our study. Previous studies also reported a stronger association of obesity and metabolic risk factors with distal colon cancer.^32^ ^44^ ^57^ ^58^ Potential explanations include differences in microbial communities,^1^ ^59^ ^60^ metabolites such as short chain fatty acids and bile acids, and carcinogenic molecular processes across the anatomic subsites of colorectal cancer contributing to divergent carcinogenesis mechanism.^61^

### Strengths and limitations of study

Strengths of this study include the prospective cohort design and high follow-up rate, which minimized recall and selection bias. Secondly, the detailed and repeated measurement of diet and other covariates enabled us to use cumulative averages of dietary intake and all the other quantitative factors to decrease measurement errors further and reduce residual confounding. The large number of cases of colorectal cancer for each subsite enabled us to examine the association of ultra-processed food with risk of colorectal cancer by anatomic subsites with sufficient statistical power.

The study also has several limitations. Firstly, owing to the study’s observational nature, residual confounding due to unmeasured confounders and measurement error of covariates cannot be ruled out. Secondly, as FFQs collect intake from only a limited number of pre-defined items representing the primary source of energy and nutrients in the study population, they cannot cover the full spectrum of ultra-processed foods consumed. Additionally, FFQs used in the three cohorts were not designed to classify food intake by levels of processing, which may lead to non-differential misclassification of the exposure. For example, nine food items lacked sufficient details in the resource documents to support their classification.^27^ We have adopted a more conservative approach assuming a lower level of processing in the primary analyses. Our sensitivity analyses using alternative classification did not materially alter the results. Thirdly, our cohort participants are US health professionals and predominantly non-Hispanic white, limiting the generalizability of our study findings. The homogeneity of our study population may have led to reduced variability in dietary intake. Stronger associations might be observed in populations with a more heterogeneous diet. Nevertheless, the associations between many risk factors and colorectal cancer risk identified in our cohorts are highly concordant with those reported in World Cancer Research Fund/American Institute of Cancer Research systematic reviews.^36^ ^55^ ^62^ ^63^

### Conclusions

In conclusion, this study found that high consumption of total ultra-processed foods in men and certain subgroups of ultra-processed foods in men and women was associated with an increased risk of colorectal cancer. The findings support the public health importance of limiting certain types of ultra-processed foods for better health outcomes in the population. Further studies are needed to better understand the potential attributes of ultra-processed foods that contribute to colorectal carcinogenesis.

## What is already known on this topic

Accumulating evidence suggests that high consumption of ultra-processed foods is associated with a higher risk of several chronic diseases
Few studies have assessed the association between ultra-processed food intake and colorectal cancer risk, and the findings are mixed owing to limitations in study design and sample sizes

## What this study adds

High consumption of total ultra-processed foods in men and certain subgroups of ultra-processed foods in men and women was associated with an increased risk of colorectal cancer
The findings support the public health importance of limiting certain types of ultra-processed foods for better health outcomes in the population

## Data Availability

No additional data available.

## Web extra

Extra material supplied by authors

Web appendix: Supplementary materials

## Notes

Contributors: LW, FFZ, and MS conceived and designed the study. MS, NK, and SLR acquired the data. LW did the statistical analysis. LW wrote the first drafted of the manuscript. All the authors critically revised the manuscript for important intellectual content and approved the final version. FFZ and MS obtained funding. FFZ and MS supervised the study and contributed equally as the senior authors. LW, FFZ, and MS are the guarantors. The corresponding author attests that all listed authors meet authorship criteria and that no others meeting the criteria have been omitted.

The lead author (the manuscript’s guarantor) affirms that the manuscript is an honest, accurate, and transparent account of the study being reported; that no important aspects of the study have been omitted; and that any discrepancies from the study as planned (and, if relevant, registered) have been explained.

Provenance and peer review: Not commissioned; externally peer reviewed.

Dissemination to participants and related patient and public communities: We plan to disseminate these findings to participants in our annual newsletter and the general public in a press release.
