# Supplementary material for: Association of ultra-processed food consumption with colorectal cancer risk among men and women: results from three prospective US cohort studies
Source: BMJ. 2022 Aug 31;378:e068921. doi: 10.1136/bmj-2021-068921 (PMC9430376; doi:10.1136/bmj-2021-068921)
Supplement: Supplementary file 1 — Web appendix: Supplementary materials [file wanl068921.ww1.pdf]

Supplemental Information

Table of Contents

Supplemental Information 1

Supplementary Methods. Target Trial Emulation of Limiting Ultra-processed Food Consumption 2

Supplementary Table A. Classification of Ultra-processed Food Subgroups for Food Items Collected in the Food Frequency Questionnaire in the Three Cohort. 3

Supplementary Table B. Percentage of Missing Values for Covariates 4

Supplementary Table C. P-values for Nonlinearity Test for Associations Between Ultra-processed food Consumption and Colorectal Cancer Risk 5

Supplementary Table D. Multivariable Adjusted Hazard Ratios of Colorectal Cancer Risk by Quintiles of Ultra-processed Food Consumption With Additional Adjustments of Body Mass Index, Diet Patterns, Diet Quality Index, and Intakes of Specific Food Groups and Nutrients[a] 6

Supplementary Table E. Hazard Ratios of Colorectal Cancer by Ultra-processed Food Consumption: Sensitivity Analysis for Alternative Definition of Undetermined Food Items [a] 8

Supplementary Table F. Hazard Ratios of Colorectal Cancer by Percentage of Energy (%E) from Ultra-processed Food Consumption 9

Supplementary Table G. Colorectal Cancer Risk by Quintiles of Ultra-processed food Consumption Among Men and Women Among Those with Less than 10 Missing FFQs 10

Supplementary Table H. Estimated Risk of Colorectal Cancer by Restricting UPF Consumption Among Men and Women in The Three Large Cohort 12

Supplementary Table I. Hazard Ratios of Colorectal Cancer Risk by Ultra-processed Food Consumption Stratified by Potential Effect Modifiers. 13

Supplementary Figure A. Participant Flow Chart 15

Supplementary Figure B. Causal Diagram for Covariates Between Ultra-processed Food Intake and Colorectal Cancer 16

Supplementary Methods. Target Trial Emulation of Limiting Ultra-processed Food Consumption

We used the target trial approach as an alternative method to evaluate the association between ultra-processed food consumption and risk of colorectal cancer in a prospective cohort study. In this approach, the parametric g-formula was used to estimate the effect of a hypothetical dietary intervention of limiting ultra-processed food consumption on colorectal cancer risk. The g-formula handles treatment-confounder feedback that occurs when the measured time-varying confounders are affected by prior dietary intake, and, under the assumptions of no unmeasured confounding and no model misspecification, estimates the risk in the study population had the hypothetical interventions been implemented.[1] Nonparametric bootstrapping with 500 samples was performed to generate percentile-based 95% CIs of the estimated risks at the time points of interest. The model also handed the competing risk of non-cancer deaths by computing the cumulative incidence of colorectal cancer, which does not assume independence of competing events. [2]

We adjusted for the following baseline covariates: age, family history of cancer, history of endoscopy and aspirin use, alcohol intake (continuous), physical activity (continuous), pack-year of smoking (continuous), BMI (continuous), and additionally for menopausal status and postmenopausal hormone use among women; and the following time-varying covariates: history of endoscopy and aspirin use, BMI, pack-year of smoking, aspirin use, physical activity, alcohol intake, menopausal status and postmenopausal hormone use(women only). All models included indicator variables for the period of follow-up, baseline covariates, and time-varying covariates measured in the previous questionnaire.

Chart 1. Emulation of a target trial of limiting ultra-processed food consumption using observational data

	Target Trail Specification	Target Trial Emulation
Eligibility criteria	No history of cancer at baseline	Same as the Target Trail Specification. We also required participants to have complete covariates and dietary intake data at baseline (e.g., missing FFQ items <70) with plausible energy intake reported (800 to 4200 kcal/d in men; 500 to 3500 kcal/d in women).
Dietary intervention	Each individual would be assigned to 1 of the following strategies for 24 years.<br>• No intervention<br>• Limiting ultra-processed food consumption to 4 servings per day	Same as the Target Trail Specification. We assumed that each 4-y dietary questionnaire accurately reflects the average diet during the previous 4-y period
Outcome	24 years of colorectal cancer, proxy colon cancer, distal colon cancer, and rectum cancer	Same as the Target Trail Specification.
Follow-up	Starts at baseline and ends at death, incomplete follow-up, or 24 y after baseline, whichever occurs first.	Same as the Target Trail Specification. Incomplete follow-up is defined as questionnaire nonresponse or incomplete responses to dietary questions.

eReferences

1. Hernán MA, Robins JM. Using Big Data to Emulate a Target Trial When a Randomized Trial Is Not Available. *American Journal of Epidemiology*. 2016;183(8):758-764. doi:10.1093/aje/kwv254
2. Young JG, Stensrud MJ, Tchetgen Tchetgen EJ, Hernán MA. A causal framework for classical statistical estimands in failure ‐ time settings with competing events. *Statistics in medicine*. 2020;39(8):1199-1236.
3. Khandpur N, Rossato S, Drouin-Chartier J-P, et al. Categorizing ultra-processed food intake in large-scale cohort studies: evidence from the Nurses' Health Studies, the Health Professionals Follow-up Study, and the Growing Up Today Study. *medRxiv*. 2021:2021.02.08.21251384. doi:10.1101/2021.02.08.21251384

Supplementary Table A. Classification of Ultra-processed Food Subgroups for Food Items Collected in the Food Frequency Questionnaire in the Three Cohort.

Ultra-processed Food subgroups	Food Items
Meat/poultry/seafood-based ready-to-eat products	Bacon; beef, pork hotdogs; chicken or turkey hotdogs; salami, bologna, processed meat sandwiches; processed meats, sausages; breaded fish cakes, pieces, sticks
Ultra-processed bread and breakfast food	Breakfast bar; cold breakfast cereal; English muffins, bagels, rolls; rye, pumpernickel bread; white bread; whole-grain bread
Packaged sweet snacks and desserts	Brownies; cake, ready-made; cookies, ready-made; doughnuts; pie, ready-made; muffins or biscuits; ready-made sweet roll, coffee cake; candy bars; chocolate bars; energy bar; high protein, low carb candy bar; applesauce; jams, jellies, preserves, honey;
Fat, condiment, and sauces	Ketchup, red chili sauce, salad dressings, mayonnaise (regular and low fat), salsa, margarine, spread butter, soy sauce, non-dairy coffee whitener, cream cheese
Sugar- or artificially- sweetened beverages	Caffeine-free Coke; Coke/Pepsi/Cola; dairy coffee drink; Hawaiian punch; Low-calorie soda, caffeine-free; low-calorie soda, Pepsi, 7-up; other carbon beverage; other low-calorie Cola with caffeine
Yogurt and dairy-based desserts	Frozen yogurt, sherbet, ice cream, ice cream, yogurt artificially sweetened; flavored yogurt without Nutrasweet
Ready-to-eat/heat mixed dishes	Pizza; chowder or cream soup; soup made with bouillon; ready-made soup from cans; French fries
Packaged savory snacks	Regular crackers; fat-free, light crackers; fat-free popcorn
Other ultra-processed foods	Nutrasweet or equal; other artificial sweeteners; Splenda

Supplementary Table B. Percentage of Missing Values for Covariates

Covariates	HPFS (No. of total observations =559,642)	NHS (No. of total observations =861,628)	NHS II (No. of total observations =1,099,212)
Ultra-processed intake	0	0	0
Age	0	0	0
White	0	0	0
Family history of colorectal cancer	0	0	0
BMI [a]	0.4%	0.1%	0.2%
Smoking status	0.4%	0.2%	0.1%
Alcohol	0		0
Physical activity	0.04%	0.1%	<0.1%
History of endoscopy	0	0	0
Regular aspirin use	0	0	0
Postmenopausal Hormone use,	NA	0	0
Postmenopausal status,	NA	0	0
Total energy intake [a]	0	0	0
Dietary fiber [a]	0	0	0
Total Calcium [a]	0	0	0
Total Vitamin D [a]	0	0	0
Processed meats [a]	<0.1%	<0.1%	<0.1%
Whole grains [a]	0	0	<0.1%
AHEI-2010 score [a]	0	0	0
Western dietary pattern [a]	0	0	0

Abbreviations: BMI, body mass index,

[a] Included in sensitivity analyses for additional adjustments.

Supplementary Table C. P-values for Nonlinearity Test for Associations Between Ultra-processed food Consumption and Colorectal Cancer Risk

Outcome	*P*-for nonlinear relationship [a]	
	Men	Women
Colorectal cancer	0.45	0.62
Proxy colon cancer	0.40	0.78
Distal colon cancer	0.20	0.14
Rectum cancer	0.81	0.51

[a] We examined the possibly non-linear relation between ultra-processed food consumption (in energy adjusted servings per day) and risk of colorectal cancer non-parametrically with restricted cubic splines in the multivariable models. The statistical significance for non-linearity was examined by the likelihood ratio test, comparing the model with only the linear term to the model with the linear and the cubic spline terms with five automatically placed knots. A P-value greater than 0.05 indicates a non-linearity relationship is not statistically significant.

Supplementary Table D. Multivariable Adjusted Hazard Ratios of Colorectal Cancer Risk by Quintiles of Ultra-processed Food Consumption With Additional Adjustments of Body Mass Index, Diet Patterns, Diet Quality Index, and Intakes of Specific Food Groups and Nutrients[a]

	Energy-adjusted Servings per Day of Ultra-Processed Food Consumption, HR (95% CI) [b]					***P*-trend** [c]
	**Quintile 1**	**Quintile 2**	**Quintile 3**	**Quintile 4**	**Quintile 5**	
**Colorectal Cancer**						
Men						
Model S1	1 [Reference]	1.20 (1.01 to 1.43)	1.02 (0.85 to 1.22)	1.13 (0.95 to 1.35)	1.24 (1.04 to 1.48)	0.02
Model S2	1 [Reference]	1.20 (1.01 to 1.43)	1.01 (0.84 to 1.22)	1.14 (0.95 to 1.37)	1.25 (1.04 to 1.49)	0.02
Model S3	1 [Reference]	1.21 (1.02 to 1.44)	1.02 (0.85 to 1.23)	1.15 (0.96 to 1.38)	1.26 (1.05 to 1.51)	0.01
Model S4	1 [Reference]	1.16 (0.97 to 1.39)	0.97 (0.80 to 1.16)	1.08 (0.90 to 1.30)	1.18 (0.98 to 1.42)	0.15
Women						
Model S1	1 [Reference]	0.89 (0.77 to 1.03)	0.94 (0.82 to 1.09)	0.97 (0.84 to 1.12)	1.01 (0.88 to 1.17)	0.87
Model S2	1 [Reference]	0.89 (0.77 to 1.03)	0.94 (0.82 to 1.09)	0.97 (0.84 to 1.12)	1.02 (0.88 to 1.18)	0.82
Model S3	1 [Reference]	0.89 (0.77 to 1.03)	0.93 (0.8 to 1.08)	0.95 (0.83 to 1.11)	1.00 (0.86 to 1.16)	0.96
Model S4	1 [Reference]	0.88 (0.76 to 1.02)	0.92 (0.80 to 1.07)	0.95 (0.82 to 1.10)	0.99 (0.85 to 1.15)	0.92
**Proximal Colon Cancer**						
Men						
Model S1	1 [Reference]	1.51 (1.12 to 2.04)	1.03 (0.74 to 1.42)	1.36 (1.00 to 1.85)	1.31 (0.96 to 1.80)	0.09
Model S2	1 [Reference]	1.53 (1.13 to 2.07)	1.05 (0.75 to 1.46)	1.40 (1.02 to 1.92)	1.37 (1.00 to 1.89)	0.05
Model S3	1 [Reference]	1.53 (1.13 to 2.07)	1.05 (0.75 to 1.46)	1.40 (1.02 to 1.91)	1.36 (0.99 to 1.87)	0.06
Model S4	1 [Reference]	1.45 (1.07 to 1.97)	0.99 (0.71 to 1.38)	1.31(0.96 to 1.80)	1.29 (0.93 to 1.78)	0.30
Women						
Model S1	1 [Reference]	0.91 (0.73 to 1.15)	1.03 (0.82 to 1.29)	0.97 (0.78 to 1.22)	1.09 (0.87 to 1.36)	0.46
Model S2	1 [Reference]	0.91 (0.72 to 1.14)	1.02 (0.82 to 1.28)	0.97 (0.77 to 1.21)	1.08 (0.86 to 1.36)	0.49
Model S3	1 [Reference]	0.90 (0.71 to 1.13)	1.01 (0.81 to 1.27)	0.95 (0.76 to 1.20)	1.07 (0.85 to 1.35)	0.58
Model S4	1 [Reference]	0.90 (0.71 to 1.13)	1.01 (0.80 to 1.27)	0.95 (0.75 to 1.20)	1.06 (0.84 to 1.35)	0.58
**Distal Colon Cancer**						
Men						
Model S1	1 [Reference]	1.12 (0.79 to 1.59)	1.14 (0.80 to 1.61)	1.09 (0.77 to 1.55)	1.64 (1.19 to 2.27)	0.003
Model S2	1 [Reference]	1.11 (0.78 to 1.57)	1.11 (0.78 to 1.57)	1.10 (0.77 to 1.57)	1.59 (1.15 to 2.22)	0.006
Model S3	1 [Reference]	1.14 (0.80 to 1.61)	1.15 (0.81 to 1.63)	1.14 (0.80 to 1.63)	1.67 (1.20 to 2.33)	0.003
Model S4	1 [Reference]	1.08 (0.76 to 1.53)	1.07 (0.75 to 1.53)	1.06 (0.74 to 1.51)	1.53 (1.09 to 2.15)	0.01
Women						
Model S1	1 [Reference]	0.76 (0.56 to 1.04)	0.96 (0.72 to 1.29)	1.04 (0.78 to 1.39)	1.05 (0.78 to 1.41)	0.75
Model S2	1 [Reference]	0.76 (0.56 to 1.04)	0.96 (0.72 to 1.29)	1.04 (0.78 to 1.40)	1.05 (0.78 to 1.42)	0.74
Model S3	1 [Reference]	0.77 (0.56 to 1.05)	0.98 (0.73 to 1.32)	1.07 (0.80 to 1.43)	1.08 (0.80 to 1.46)	0.61
Model S4	1 [Reference]	0.78 (0.57 to 1.06)	0.99 (0.73 to 1.33)	1.08 (0.80 to 1.46)	1.10 (0.81 to 1.49)	0.25
**Rectal Cancer**						

	Energy-adjusted Servings per Day of Ultra-Processed Food Consumption, HR (95% CI) [b]					*P*-trend [c]
	**Quintile 1**	**Quintile 2**	**Quintile 3**	**Quintile 4**	**Quintile 5**	
Men						
Model S1	1 [Reference]	1.19 (0.82 to 1.74)	1.12 (0.76 to 1.64)	1.01 (0.68 to 1.50)	1.02 (0.69 to 1.51)	0.93
Model S2	1 [Reference]	1.16 (0.79 to 1.69)	1.08 (0.73 to 1.58)	0.96 (0.64 to 1.44)	0.97 (0.64 to 1.45)	0.87
Model S3	1 [Reference]	1.19 (0.81 to 1.73)	1.11 (0.75 to 1.64)	1.00 (0.67 to 1.49)	1.01 (0.67 to 1.51)	0.97
Model S4	1 [Reference]	1.16 (0.79 to 1.71)	1.07 (0.72 to 1.59)	0.96 (0.64 to 1.45)	0.97 (0.64 to 1.47)	0.55
Women						
Model S1	1 [Reference]	1.10 (0.81 to 1.49)	0.86 (0.62 to 1.19)	0.94 (0.68 to 1.29)	1.05 (0.76 to 1.44)	0.76
Model S2	1 [Reference]	1.10 (0.81 to 1.50)	0.87 (0.62 to 1.20)	0.95 (0.69 to 1.31)	1.06 (0.77 to 1.47)	0.71
Model S3	1 [Reference]	1.09 (0.80 to 1.48)	0.85 (0.61 to 1.18)	0.92 (0.66 to 1.28)	1.02 (0.74 to 1.42)	0.89
Model S4	1 [Reference]	1.06 (0.78 to 1.45)	0.83 (0.59 to 1.15)	0.90 (0.65 to 1.25)	1.01 (0.72 to 1.41)	0.76

Abbreviations: BMI, body mass index; HR, hazard ratio; 95% CI: 95% confidence interval.

[a]: All models (Models S1-S4) were adjusted for covariates as in the main models (Table 2), including age, calendar year of the current questionnaire, race, family history of cancer, history of endoscopy, alcohol intake (in g/day: <5, 5-9.9, 10-14.9, 15-29.9, or ≥30), physical activity (in metabolic equivalent-hours/week: <3, 3-8.9, 9-17.9, 18-26.9, or ≥27), status and pack-years of smoking(never, past smoker with pack-years <5, past smoker with pack-years ≥5, current smoker with pack-years <20, current smoker with pack-years ≥20), total caloric intake (quintiles), regular aspirin use, and additionally for menopausal status and postmenopausal hormone use in women. Model S1 additionally adjusted for BMI; Model S2 additional adjusted for factor scores of the western dietary pattern derived from principal component analyses; Model S3 additionally adjusted for Alternative Health Eating Index (AHEI)-2010; Model s4 additionally adjusted for processed meats, fruits, vegetables, whole grains, dietary calcium, and dietary vitamin D.

[b] The median (range) for each quintile among women: quintile 1= 3.9 (0.1-5.1), quintile 2=5.2 (4.0-6.2), quintile 3=6.2 (5.0-7.3), quintile 4=7.3 (5.9-8.9), quintile 5=9.4 (7.2-42.6); among men: quintile 1=3.8(0.1-4.8), quintile 2=5.2 (4.1-6.0), quintile 3=6.3(5.2-7.1), quintile 4=7.5(6.3-8.5), quintile 5=9.6 (7.9-28.0). As cycle-specific quintiles were generated, overlapping ranges of adjunct quintiles exist.

[c]: *P-value* for linear trend of colorectal cancer risk across quintiles of ultra-processed food consumption was obtained by assigning the quintile medians to each participant in the quintile as an ordinal variable, adjusted for the same set of covariates as above.

Supplementary Table E. Hazard Ratios of Colorectal Cancer by Ultra-processed Food Consumption: Sensitivity Analysis for Alternative Definition of Undetermined Food Items [a]

Disease	Energy-adjusted Servings per Day of Ultra-Processed Food Consumption, HR (95% CI)					*P*-trend [c]
	Quintile 1	Quintile 2	Quintile 3	Quintile 4	Quintile 5	
**Colorectal Cancer**						
Men						
Age-adjusted model	1 [Reference]	1.07 (0.89 to 1.27)	0.97 (0.81 to 1.16)	1.11 (0.93 to 1.32)	1.23 (1.04 to 1.46)	0.01
Multivariable-adjusted model [b]	1 [Reference]	1.09 (0.92 to 1.30)	1.01 (0.84 to 1.20)	1.16 (0.97 to 1.38)	1.29 (1.08 to 1.53)	0.003
Women						
Age-adjusted model	1 [Reference]	0.93 (0.80 to 1.07)	0.97 (0.85 to 1.12)	0.98 (0.85 to 1.13)	1.14 (0.99 to 1.31)	0.03
Multivariable-adjusted model [b]	1 [Reference]	0.92 (0.80 to 1.07)	0.96 (0.83 to 1.11)	0.96 (0.83 to 1.11)	1.09 (0.94 to 1.25)	0.15
**Proximal Colon Cancer**						
Men						
Age-adjusted model	1 [Reference]	1.43 (1.06 to 1.93)	1.14 (0.83 to 1.56)	1.31 (0.97 to 1.79)	1.38 (1.01 to 1.87)	0.11
Multivariable-adjusted model [b]	1 [Reference]	1.44 (1.07 to 1.95)	1.15 (0.84 to 1.58)	1.33 (0.97 to 1.82)	1.40 (1.02 to 1.91)	0.10
Women						
Age-adjusted model	1 [Reference]	0.98 (0.79 to 1.23)	1.01 (0.81 to 1.25)	0.92 (0.73 to 1.15)	1.19 (0.96 to 1.48)	0.16
Multivariable-adjusted model [b]	1 [Reference]	0.97 (0.78 to 1.21)	1.00 (0.80 to 1.25)	0.90 (0.72 to 1.14)	1.13 (0.90 to 1.41)	0.36
**Distal Colon Cancer**						
Men						
Age-adjusted model	1 [Reference]	1.08 (0.77 to 1.53)	0.96 (0.67 to 1.36)	1.25 (0.90 to 1.75)	1.62 (1.18 to 2.22)	<0.001
Multivariable-adjusted model [b]	1 [Reference]	1.12 (0.79 to 1.57)	1.01 (0.70 to 1.43)	1.31 (0.93 to 1.83)	1.72 (1.25 to 2.37)	<0.001
Women						
Age-adjusted model	1 [Reference]	0.88 (0.65 to 1.20)	1.18 (0.89 to 1.58)	1.17 (0.87 to 1.56)	1.22 (0.91 to 1.63)	0.06
Multivariable-adjusted model [b]	1 [Reference]	0.88 (0.64 to 1.20)	1.17 (0.88 to 1.57)	1.15 (0.85 to 1.54)	1.18 (0.87 to 1.59)	0.11
**Rectal Cancer**						
Men						
Age-adjusted model	1 [Reference]	0.95 (0.64 to 1.39)	1.12 (0.77 to 1.62)	0.91 (0.62 to 1.35)	1.06 (0.73 to 1.55)	0.81
Multivariable-adjusted model [b]	1 [Reference]	0.99 (0.67 to 1.46)	1.18 (0.81 to 1.72)	0.97 (0.65 to 1.45)	1.11 (0.76 to 1.63)	0.65
Women						
Age-adjusted model	1 [Reference]	0.98 (0.72 to 1.32)	0.72 (0.52 to 1.00)	0.90 (0.66 to 1.23)	1.15 (0.86 to 1.55)	0.38
Multivariable-adjusted model [b]	1 [Reference]	0.98 (0.72 to 1.33)	0.71 (0.51 to 0.99)	0.89 (0.65 to 1.22)	1.12 (0.83 to 1.52)	0.49

Abbreviations: HR, hazard ratio; 95%CI: 95% confidence interval.

[a]: In alternative definition, several food items that lack sufficient information to determine their categorization [3] and were previously classified as non-ultra-processed foods were reclassified as ultra-processed foods, including “Popcorn”, “Soy milk”, “Chicken or turkey sandwich”, “Pancakes or waffles”, “Pie, home-baked or ready-made”, “Beef, Pork, Lamb Sandwich”, and “Tomato Sauce”.

[b]: All multivariable-adjusted models were adjusted for the following potential confounding variables: age (in years), calendar year of the current questionnaire, race, family history of cancer, history of endoscopy, total alcohol intake (in g/day: <5, 5-9.9, 10-14.9, 15-29.9, or ≥30), physical activity (in metabolic equivalent-hours/week: <3, 3-8.9, 9-17.9, 18-26, or ≥27), smoking status and pack-years of smoking (never, past smoker with pack-years <5, past smoker with pack-years ≥5, current smoker with pack-years <20, current smoker with pack-years 20), total caloric intake (quintiles), regular aspirin use, and additionally for menopausal status and postmenopausal hormone use in women.

[c]: *P-value* for linear trend of colorectal cancer risk across quintiles of ultra-processed food consumption was obtained by assigning the quintile medians to each participant in the quintile as an ordinal variable, adjusted for the same set of covariates as above.

Supplementary Table F. Hazard Ratios of Colorectal Cancer by Percentage of Energy (%E) from Ultra-processed Food Consumption

Disease	%E of Ultra-processed foods, HR (95% CI) [a]					*P*-trend [c]
	Quintile 1	Quintile 2	Quintile 3	Quintile 4	Quintile 5	
**Colorectal Cancer**						
Men						
Age-adjusted model	1 [Reference]	1.06 (0.90 to 1.26)	1.01 (0.84 to 1.20)	1.04 (0.87 to 1.23)	1.17 (0.98 to 1.39)	0.13
Multivariable-adjusted model [b]	1 [Reference]	1.08 (0.91 to 1.28)	1.04 (0.88 to 1.24)	1.09 (0.91 to 1.30)	1.24 (1.03 to 1.48)	0.03
Women						
Age-adjusted model	1 [Reference]	1.03 (0.89 to 1.18)	1.00 (0.87 to 1.16)	1.03 (0.89 to 1.19)	1.15 (0.99 to 1.32)	0.08
Multivariable-adjusted model [b]	1 [Reference]	1.03 (0.89 to 1.19)	0.99 (0.86 to 1.15)	1.01 (0.88 to 1.17)	1.10 (0.95 to 1.28)	0.28
Proximal Colon Cancer						
Men						
Age-adjusted model	1 [Reference]	1.24 (0.93 to 1.64)	0.88 (0.65 to 1.20)	1.14 (0.85 to 1.53)	1.12 (0.83 to 1.52)	0.65
Multivariable-adjusted model [b]	1 [Reference]	1.23 (0.93 to 1.63)	0.89 (0.65 to 1.21)	1.16 (0.86 to 1.56)	1.14 (0.84 to 1.56)	0.55
Women						
Age-adjusted model	1 [Reference]	1.21 (0.97 to 1.50)	1.12 (0.90 to 1.40)	1.06 (0.84 to 1.33)	1.23 (0.98 to 1.54)	0.25
Multivariable-adjusted model [b]	1 [Reference]	1.21 (0.97 to 1.51)	1.11 (0.88 to 1.39)	1.05 (0.83 to 1.32)	1.19 (0.94 to 1.51)	0.42
Distal Colon Cancer						
Men						
Age-adjusted model	1 [Reference]	1.27 (0.91 to 1.77)	1.27 (0.90 to 1.77)	1.22 (0.86 to 1.72)	1.63 (1.17 to 2.27)	0.008
Multivariable-adjusted model [b]	1 [Reference]	1.30 (0.93 to 1.82)	1.34 (0.95 to 1.88)	1.31 (0.93 to 1.86)	1.78 (1.26 to 2.50)	0.002
Women						
Age-adjusted model	1 [Reference]	0.80 (0.60 to 1.08)	0.85 (0.63 to 1.13)	0.91 (0.69 to 1.22)	1.11 (0.84 to 1.47)	0.31
Multivariable-adjusted model [b]	1 [Reference]	0.80 (0.60 to 1.07)	0.84 (0.63 to 1.13)	0.89 (0.66 to 1.19)	1.05 (0.78 to 1.41)	0.52
Rectal Cancer						
Men						
Age-adjusted model	1 [Reference]	0.72 (0.49 to 1.05)	0.89 (0.62 to 1.27)	0.82 (0.56 to 1.18)	0.90 (0.62 to 1.31)	0.75
Multivariable-adjusted model [b]	1 [Reference]	0.75 (0.51 to 1.09)	0.94 (0.65 to 1.35)	0.87 (0.59 to 1.26)	0.96 (0.66 to 1.41)	0.99
Women						
Age-adjusted model	1 [Reference]	1.24 (0.90 to 1.70)	1.14 (0.82 to 1.57)	1.23 (0.89 to 1.69)	1.24 (0.90 to 1.72)	0.25
Multivariable-adjusted model [b]	1 [Reference]	1.25 (0.91 to 1.72)	1.13 (0.82 to 1.57)	1.24 (0.89 to 1.71)	1.23 (0.88 to 1.73)	0.30

Abbreviations: HR, hazard ratio; 95% CI: 95% confidence interval;%E, percentage of energy.

[a]: The median and range for each quintile among men were quintile 1: 18% (0.5%-23%); quintile 2: 25% (20%-28%); quintile 3: 29%(25%-33%); quintile 4: 34%(30%-38%); quintile 5: 41% (36%-86); range for each quintile among women were quintile 1: 22%(0.1-27.9%); quintile 2: 29%(24%-33%); quintile 3: 33%(29%-37%); quintile 4: 38%(34%-44%); quintile 5: 46%(40%-94%). Ranges for adjunct quintiles overlapped as quintiles were generated for each survey cycle separately.

[b] : All multivariable-adjusted models were adjusted for the following potential confounding variables: age, calendar year of the current questionnaire, race, family history of cancer, history of endoscopy, total alcohol intake (in g/day: <5, 5-9.9, 10-14.9, 15-29.9, or ≥30), physical activity (in metabolic equivalent-hours/week: <3, 3-8.9, 9-17.9, 18-26.9, or ≥27), smoking status and pack-years of smoking(never, past smoker with pack-years <5, past smoker with pack-years ≥5, current smoker with pack-years <20, current smoker with pack-years≥20), total caloric intake (quintiles), regular aspirin use, and additionally for menopausal status and postmenopausal hormone use in women.

[c]: *P-value* for linear trend of colorectal cancer risk across quintiles of ultra-processed food consumption was obtained by assigning the quintile medians to each participant in the quintile as an ordinal variable, adjusted for the same set of covariates as above.

Supplementary Table G. Colorectal Cancer Risk by Quintiles of Ultra-processed food Consumption Among Men and Women Among Those with Less than 10 Missing FFQs

	Energy Adjusted Servings Per Day of Ultra-processed Food intake, HR (95% CI) [a]					*P*-trend [b]	Continuous, HR (95% CI)
	Quintile 1	Quintile 2	Quintile 3	Quintile 4	Quintile 5		
**Colorectal Cancer**							
Men (HPFS)							
Cases/person-years	218/192270	252/204887	220/208124	248/208397	269/204519		
Age-adjusted model	1 [Reference]	1.14 (0.95 to 1.37)	0.97 (0.80 to 1.17)	1.10 (0.92 to 1.32)	1.20 (1.00 to 1.43)	0.08	1.03 (1.00 to 1.06)
Multivariable-adjusted model [a]	1 [Reference]	1.18 (0.98 to 1.41)	1.01 (0.84 to 1.23)	1.16 (0.96 to 1.39)	1.25 (1.04 to 1.50)	0.03	1.03 (1.00 to 1.06)
Women (NHS+NHS II)							
Cases/person-years	367/741757	354/784013	376/787581	385/777237	396/741142		
Age-adjusted model	1 [Reference]	0.91 (0.79 to 1.06)	0.96 (0.83 to 1.11)	1.00 (0.86 to 1.15)	1.09 (0.94 to 1.25)	0.09	1.02 (1.00 to 1.05)
Multivariable-adjusted model [a]	1 [Reference]	0.91 (0.78 to 1.05)	0.95 (0.83 to 1.10)	0.98 (0.84 to 1.13)	1.04 (0.90 to 1.21)	0.30	1.01 (0.99 to 1.04)
**Proximal Colon Cancer**							
Men (HPFS)							
Cases	63	98	71	91	86		
Age-adjusted model	1 [Reference]	1.53 (1.11 to 2.09)	1.07 (0.77 to 1.50)	1.39 (1.01 to 1.91)	1.31 (0.95 to 1.82)	0.27	1.03 (0.98 to 1.08)
Multivariable-adjusted model [a]	1 [Reference]	1.54 (1.12 to 2.11)	1.09 (0.77 to 1.53)	1.41 (1.02 to 1.94)	1.33 (0.96 to 1.85)	0.25	1.03 (0.98 to 1.08)
Women (NHS+NHS II)							
Cases	145	146	163	155	168		
Age-adjusted model	1 [Reference]	0.95 (0.76 to 1.20)	1.06 (0.84 to 1.32)	1.01 (0.81 to 1.27)	1.17 (0.93 to 1.46)	0.14	1.03 (0.99 to 1.08)
Multivariable-adjusted model [a]	1 [Reference]	0.93 (0.74 to 1.18)	1.04 (0.83 to 1.30)	0.99 (0.79 to 1.25)	1.11 (0.89 to 1.40)	0.28	1.02 (0.98 to 1.07)
**Distal Colon Cancer**							
Men (HPFS)							
Cases	59	61	66	63	97		
Age-adjusted model	1 [Reference]	1.02 (0.71 to 1.46)	1.07 (0.76 to 1.53)	1.04 (0.73 to 1.48)	1.58 (1.14 to 2.18)	0.003	1.08 (1.03 to 1.14)
Multivariable-adjusted model [a]	1 [Reference]	1.06 (0.74 to 1.52)	1.14 (0.80 to 1.62)	1.11 (0.77 to 1.58)	1.68 (1.21 to 2.34)	0.001	1.09 (1.04 to 1.15)
Women (NHS+NHS II)							
Cases	89	71	91	98	98		
Age-adjusted model	1 [Reference]	0.76 (0.56 to 1.04)	0.97 (0.72 to 1.30)	1.05 (0.79 to 1.40)	1.11 (0.83 to 1.48)	0.12	1.04 (0.99 to 1.10)
Multivariable-adjusted model [a]	1 [Reference]	0.77 (0.56 to 1.05)	0.97 (0.72 to 1.30)	1.04 (0.78 to 1.39)	1.08 (0.81 to 1.45)	0.19	1.04 (0.98 to 1.10)
**Rectal Cancer**							
Men (HPFS)							
Cases	49	54	50	49	47		
Age-adjusted model	1 [Reference]	1.07 (0.73 to 1.58)	0.97 (0.65 to 1.44)	0.96 (0.64 to 1.42)	0.93 (0.62 to 1.39)	0.56	0.98 (0.92 to 1.05)
Multivariable-adjusted model [a]	1 [Reference]	1.13 (0.77 to 1.67)	1.04 (0.70 to 1.55)	1.03 (0.69 to 1.54)	0.98 (0.65 to 1.47)	0.75	0.99 (0.93 to 1.06)
Women (NHS+NHS II)							
Cases	74	87	69	75	81		
Age-adjusted model	1 [Reference]	1.11 (0.81 to 1.51)	0.88 (0.63 to 1.22)	0.97 (0.70 to 1.34)	1.11 (0.81 to 1.52)	0.72	1.01 (0.95 to 1.07)
Multivariable-adjusted model [a]	1 [Reference]	1.12 (0.82 to 1.53)	0.88 (0.63 to 1.22)	0.95 (0.69 to 1.32)	1.09 (0.79 to 1.51)	0.83	1.01 (0.95 to 1.07)

Abbreviations: HR, hazard ratio; 95% CI: 95% confidence interval.

[a] Multivariable-adjusted model was adjusted for the following potential confounding variables: age, calendar year of the current questionnaire, race, family history of cancer, history of endoscopy, total alcohol intake (in g/day: <5, 5-9.9, 10-14.9, 15-29.9, or ≥30), physical activity (in metabolic equivalent-hours/week: <3, 3-8.9, 9-17.9, 18-26, or ≥27), smoking status and pack-years of smoking(never, past smoker with pack-years <5, past smoker with pack-years ≥5, current smoker with pack-years <20, current smoker with pack-years ≥20), total caloric intake (quintiles), regular aspirin use, and additionally for menopausal status and postmenopausal hormone use in women.

[b] *P-value* for linear trend of colorectal cancer risk across quintiles of ultra-processed food consumption was obtained by assigning the quintile medians to each participant in the quintile as an ordinal variable, adjusted for the same set of covariates as above.
[d] *P*-heterogeneity between sex for the multivariable-adjusted model.

Supplementary Table H. Estimated Risk of Colorectal Cancer by Restricting UPF Consumption Among Men and Women in The Three Large Cohort

	**Men (N=46341)**			**Women[b] (N=159907)**		
	**Predicted risk during follow-up[a] (95% CI), %**	**Risk difference (95% CI), %**	**Risk ratio (95% CI), %**	**Predicted risk during follow-up[c] (95% CI), %**	**Risk difference (95% CI), %**	**Risk ratio (95% CI), %**
Risk of colorectal cancer						
No intervention	2.80(2.62 to 2.98)	0 (reference)	1 (reference)	1.32 (1.25 to 1.42)	0 (reference)	1 (reference)
Intervention: limit UPF to 4 servings per day	2.64(2.41 to 2.89)	-0.16 (-0.32 to 0.01)	0.94 (0.89 to 1.00)	1.28 (1.21 to 1.38)	-0.04 (-0.16 to 0.08)	0.97 (0.88 to 1.06)
Risk of proxy colon cancer						
No intervention	1.09(0.94 to 1.35)	0 (reference)	1 (reference)	0.55 (0.51 to 0.6)	0 (reference)	1 (reference)
Intervention: limit UPF to 4 servings per day	1.05(0.88 to 1.33)	-0.03 (-0.15 to 0.08)	0.97 (0.87 to 1.07)	0.51 (0.44 to 0.57)	-0.04 (-0.10 to 0.03)	0.92 (0.82 to 1.05)
Risk of distal colon cancer						
No intervention	0.84(0.73 to 1.07)	0 (reference)	1 (reference)	0.31 (0.27 to 0.35)	0 (reference)	1 (reference)
Intervention: limit UPF to 4 servings per day	0.69(0.56 to 0.92)	-0.15 (-0.24 to -0.05)	0.82 (0.72 to 0.94)	0.30 (0.24 to 0.35)	-0.01 (-0.07 to 0.05)	0.97 (0.78 to 1.15)
Risk of rectum cancer						
No intervention	0.58(0.51 to 0.66)	0 (reference)	1 (reference)	0.28 (0.23 to 0.34)	0 (reference)	1 (reference)
Intervention: limit UPF to 4 servings per day	0.62(0.51 to 0.74)	0.04 (-0.04 to 0.13)	1.06 (0.92 to 1.23)	0.27 (0.23 to 0.32)	-0.01 (-0.1 to 0.05)	0.97 (0.73 to 1.19)

Abbreviations: UPF, ultra-processed foods; CI, confidence interval.

Estimates are based on the parametric g-formula with baseline covariates: baseline age, family history of cancer, history of endoscopy and aspirin use, intake (continuous), physical activity (continuous), pack-year of smoking (continuous), and BMI (continuous), menopausal status (NHS/NHSII) and postmenopausal hormone use (NHS/NHSII); and time-varying covariates: pack-year of smoking, aspirin, physical activity, history of endoscopy, alcohol, BMI, and postmenopausal hormone use (NHS/NHSII).

[a.] The length of follow-up for men in HPFS is 28 years.

[b]. Including women from NHS and NHS II

[c.] The length of follow-up for women in NHS is 28 years and 24 years in NHS II.

Supplementary Table I. Hazard Ratios of Colorectal Cancer Risk by Ultra-processed Food Consumption Stratified by Potential Effect Modifiers.

Subgroups	No. of cases	Energy adjusted servings per day of ultra-processed food consumption- HR (95% CI) [a]					*P*-Trend [b]	P-Heterogeneity [c]
		Quintile 1	Quintile 2	Quintile 3	Quintile 4	Quintile 5		
**Family history of colorectal cancer**								
Men								0.82
Yes	280	1 [Reference]	1.73 (1.18 to 2.54)	1.16 (0.77 to 1.76)	1.34 (0.89 to 2.01)	1.50 (1.01 to 2.24)	0.24	
No	1014	1 [Reference]	1.11 (0.91 to 1.35)	1.01 (0.83 to 1.24)	1.14 (0.93 to 1.39)	1.23 (1.01 to 1.50)	0.04	
Women								0.29
Yes	553	1 [Reference]	1.02 (0.76 to 1.38)	0.91 (0.67 to 1.24)	1.07 (0.80 to 1.44)	1.11 (0.82 to 1.49)	0.44	
No	1767	1 [Reference]	0.87 (0.74 to 1.03)	0.96 (0.82 to 1.13)	0.95 (0.81 to 1.12)	1.00 (0.85 to 1.17)	0.65	
**BMI**								
Men								0.99
<25.0	502	1 [Reference]	1.17 (0.89 to 1.54)	1.02 (0.77 to 1.36)	1.24 (0.94 to 1.63)	1.31 (0.99 to 1.74)	0.054	
25.0~29.9	637	1 [Reference]	1.17 (0.91 to 1.51)	1.01 (0.77 to 1.31)	1.10 (0.85 to 1.42)	1.16 (0.90 to 1.50)	0.40	
≥30.0	155	1 [Reference]	1.36 (0.78 to 2.39)	1.03 (0.57 to 1.87)	0.93 (0.52 to 1.65)	1.25 (0.73 to 2.14)	0.75	
Women								0.99
<25.0	1090	1 [Reference]	0.96 (0.79 to 1.17)	0.83 (0.68 to 1.02)	0.85 (0.69 to 1.05)	1.05 (0.85 to 1.28)	0.97	
25.0~29.9	766	1 [Reference]	0.83 (0.64 to 1.08)	1.05 (0.82 to 1.35)	0.98 (0.76 to 1.26)	0.93 (0.72 to 1.21)	0.99	
≥30.0	464	1 [Reference]	0.84 (0.57 to 1.24)	1.09 (0.76 to 1.57)	1.21 (0.86 to 1.72)	1.10 (0.78 to 1.57)	0.23	
**Physical activity**								
Men								0.07
<15	465	1 [Reference]	1.18 (0.88 to 1.57)	0.82 (0.60 to 1.12)	1.04 (0.78 to 1.40)	1.03 (0.77 to 1.37)	0.93	
≥15	829	1 [Reference]	1.25 (1.00 to 1.56)	1.18 (0.94 to 1.48)	1.25 (1.00 to 1.57)	1.47 (1.18 to 1.84)	0.001	
Women								0.11
<15	1376	1 [Reference]	0.91 (0.74 to 1.12)	0.96 (0.79 to 1.17)	1.02 (0.84 to 1.23)	1.08 (0.90 to 1.31)	0.15	
≥15	944	1 [Reference]	0.91 (0.74 to 1.12)	0.96 (0.78 to 1.19)	0.93 (0.75 to 1.16)	0.93 (0.74 to 1.18)	0.63	
**Smoking Status**								
Men								0.63
Never	521	1 [Reference]	1.29 (1.00 to 1.68)	0.95 (0.71 to 1.26)	1.12 (0.85 to 1.48)	1.26 (0.95 to 1.66)	0.29	
0-20 pack-year	286	1 [Reference]	1.28 (0.87 to 1.88)	1.37 (0.94 to 2.00)	1.38 (0.94 to 2.03)	1.48 (1.01 to 2.17)	0.054	

>20 pack-year	419	1 [Reference]	1.12 (0.81 to 1.54)	0.92 (0.66 to 1.29)	1.00 (0.72 to 1.38)	1.17 (0.87 to 1.59)	0.36	
Women								0.99
Never	946	1 [Reference]	1.03 (0.82 to 1.28)	1.03 (0.83 to 1.28)	1.04 (0.83 to 1.29)	1.02 (0.81 to 1.28)	0.89	
0-20 pack-year	586	1 [Reference]	0.80 (0.61 to 1.04)	0.82 (0.63 to 1.08)	0.78 (0.59 to 1.03)	0.95 (0.72 to 1.25)	0.79	
>20 pack-year	741	1 [Reference]	0.82 (0.61 to 1.10)	1.01 (0.76 to 1.32)	1.10 (0.84 to 1.44)	1.07 (0.82 to 1.39)	0.19	
**Alternative health eating index**								
Men								0.98
Tertile 1	429	1 [Reference]	1.17 (0.81 to 1.70)	0.81 (0.55 to 1.19)	1.03 (0.72 to 1.48)	1.16 (0.82 to 1.65)	0.33	
Tertile 2	428	1 [Reference]	0.90 (0.65 to 1.26)	1.19 (0.86 to 1.63)	1.15 (0.83 to 1.58)	1.14 (0.83 to 1.58)	0.21	
Tertile 3	437	1 [Reference]	1.45 (1.13 to 1.87)	0.97 (0.72 to 1.31)	1.19 (0.88 to 1.61)	1.34 (0.97 to 1.83)	0.21	
Women								0.99
Tertile 1	747	1 [Reference]	0.82 (0.60 to 1.12)	0.81 (0.60 to 1.08)	0.83 (0.63 to 1.11)	0.88 (0.66 to 1.16)	0.81	
Tertile 2	816	1 [Reference]	0.77 (0.58 to 1.00)	1.00 (0.78 to 1.28)	1.03 (0.80 to 1.32)	1.03 (0.79 to 1.33)	0.22	
Tertile 3	757	1 [Reference]	1.07 (0.87 to 1.32)	0.97 (0.77 to 1.22)	0.99 (0.77 to 1.26)	1.03 (0.78 to 1.35)	0.94	

Abbreviations: HR, hazard ratio; 95% CI: 95% confidence interval; Q: Quintile

[a]: Hazard ratios were estimated from time-varying Cox models adjusted for the following potential confounding variables (if not the stratification factor): age, calendar year of the current questionnaire, race, family history of cancer, history of endoscopy, total alcohol intake (in g/day: <5, 5-9.9, 10-14.9, 15-29.9, or ≥30), physical activity (in metabolic equivalent-hours/week: <3, 3-8.9, 9-17.9, 18-26.9, or ≥27), smoking status and pack-years of smoking(never, past smoker with pack-years <5, past smoker with pack-years ≥5, current smoker with pack-years <20, current smoker with pack-years ≥20), total caloric intake (quintiles), regular aspirin use, and additionally for menopausal status and postmenopausal hormone use in women.

[b]: P-value for linear trend of colorectal cancer risk across quintiles of ultra-processed food consumption was obtained by assigning the quintile medians to each participant in the quintile as an ordinal variable, adjusted for the same set of covariates as above.

[c]: Statistical significance for heterogeneity between subgroups (i.e. effect modification) was tested using Likelihood Ratio tests comparing models with and without the interaction term of ultra-processed food consumption and the potential modifier variable,

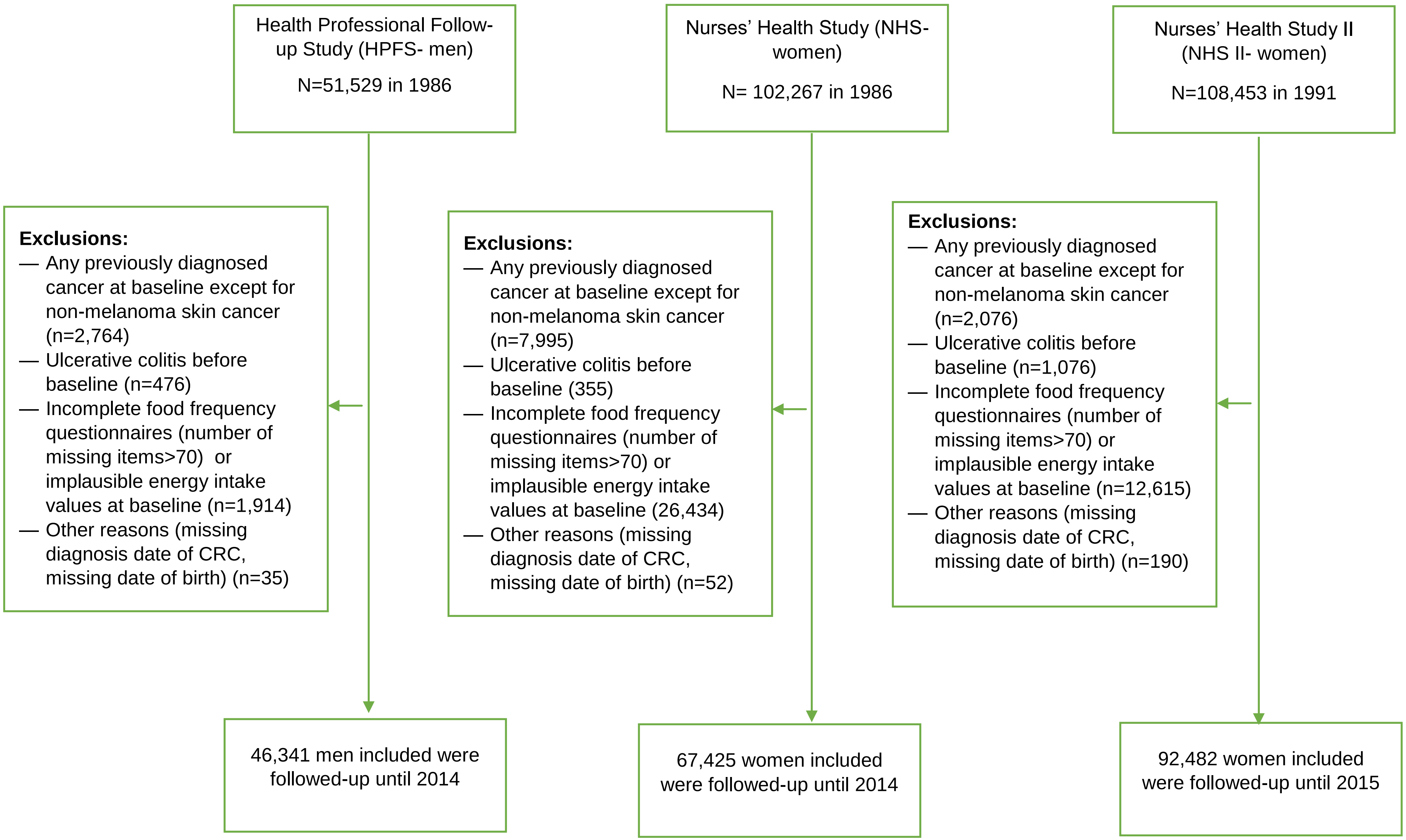

Supplementary Figure A. Participant Flow Chart

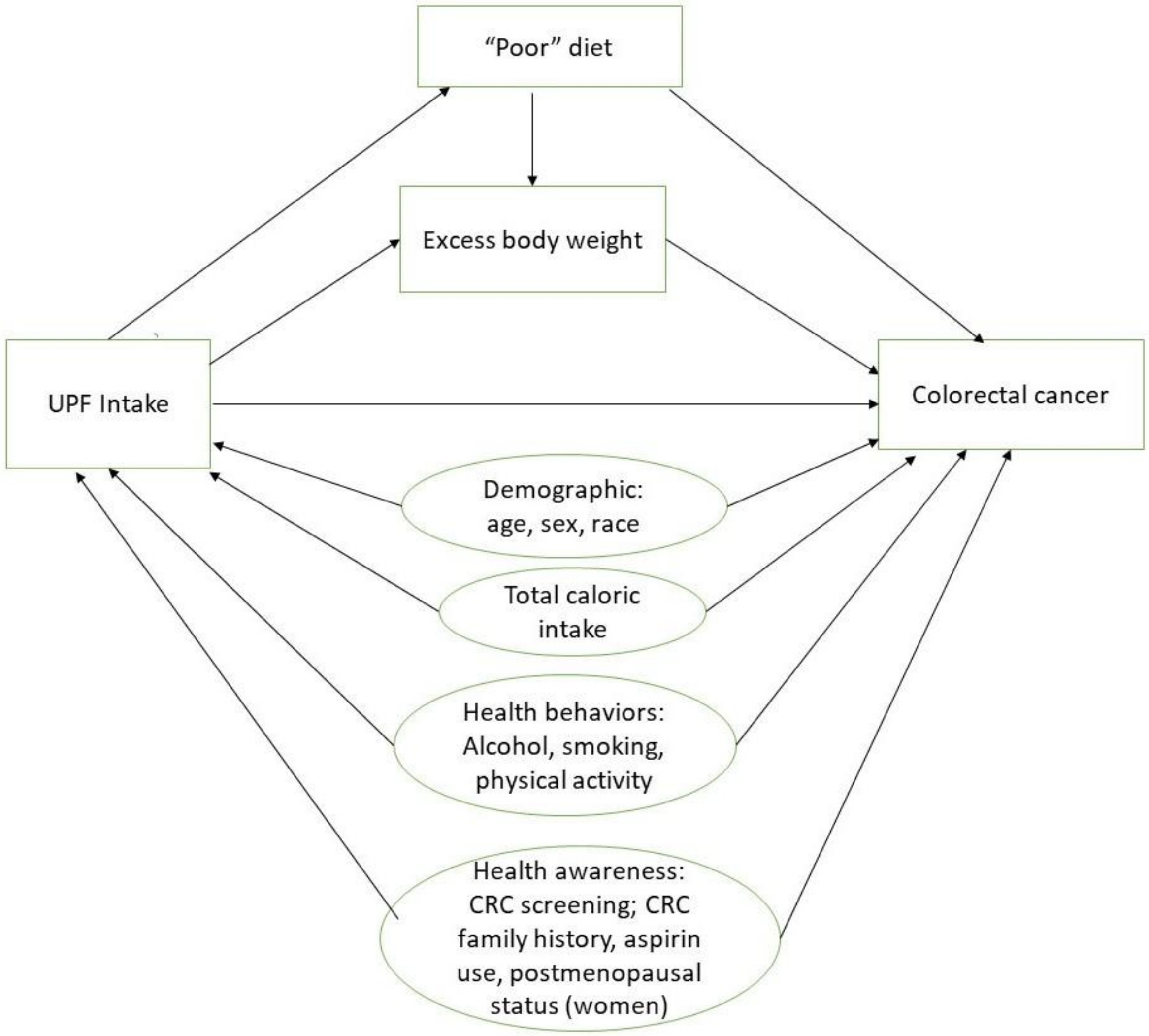

Supplementary Figure B. Causal Diagram for Covariates Between Ultra-processed Food Intake and Colorectal Cancer
Abbreviation: UPF: ultra-processed food.
Note: For the causal links between UPF intake and BMI, and between UPF and poor diet, the direction of arrows can be reversed. For example, lack of attention to healthy weight could lead to high BMI as well as high UPF intake; Also, UPF intake might be correlated with poor diet, due to low health consciousness. We therefore additionally adjusted for BMI, and indicators of poor diet in sperate models (Model S1-S4). We may not be able to present all possible causal relationships amongst the covariates in the diagram due to the lack of complete knowledge of the causal relationships.
